# Exploring the Associations Between *CHRNA5* and *IREB2* Gene Polymorphisms and COPD in the Kazakhstan Population

**DOI:** 10.3390/biomedicines13092260

**Published:** 2025-09-13

**Authors:** Almira Akparova, Gaukhar Kurmanova, Gulzhan Trimova, Yeldar Ashirbekov, Diana Nigmatova, Balkiya Abdrakhmanova, Zhanar Mussagulova, Gulzhana Idrisova, Anarkul Kulembayeva, Almagul Kurmanova

**Affiliations:** 1 Faculty of Medicine and Healthcare, Al-Farabi Kazakh National University, Almaty 050040, Kazakhstan; akparova-a@yandex.kz (A.A.); trimova@gmail.com (G.T.); shakizadakizi@mail.ru (Z.M.); gulzhana.idrissova@gmail.com (G.I.); dr_kulembayeva@mail.ru (A.K.); 2 Laboratory of Structural and Functional Genomics, M. Aitkhozhin Institute of Molecular Biology and Biochemistry, 86, Dosmukhamedov Street, Almaty 050012, Kazakhstan; eldarasher@mail.ru; 3 Respiratory Center, City Clinical Hospital No 1, Almaty 050006, Kazakhstan; nigmatova_diana@mail.ru; 4 Department of General Biology and Genomics, L.N. Gumilyov Eurasian National University, 2, Satpayev Str., Astana 010008, Kazakhstan; moldir-arujan@mail.ru

**Keywords:** COPD, Kazakhstan, single-nucleotide polymorphisms, *IREB2*, *CHRNA5*

## Abstract

**Background/Objectives**: Chronic obstructive pulmonary disease (COPD) is a progressive inflammatory lung disease characterized by irreversible airway obstruction. This study aims to investigate the associations between COPD and its phenotypes with polymorphic variants of the *IREB2* and *CHRNA5* genes in the Kazakhstan population. **Methods**: A case–control study was conducted involving 265 COPD patients and 267 controls. Genotyping of the *IREB2* polymorphisms *rs13180* and *rs2568494*, as well as *CHRNA5 rs16969968* polymorphism, was performed using real-time polymerase chain reactions (Real-Time PCRs). **Results**: A higher frequency of the AA genotype of the *IREB2 rs2568494* polymorphism was identified in COPD patients with moderate to very severe airflow obstruction (Chronic Obstructive Lung Disease (GOLD) stages II, III, and IV), with an odds ratio of 0.69 (95% CI = 0.23–2.10; Padj = 0.03). The *IREB2 rs13180* polymorphism was significantly more frequent or prevalent in smokers and showed a correlation with FEV_1_ (forced expiratory volume in one second) (β = 7.79, SE = 2.98, *p* = 0.01) and FEV_1_/ FVC (forced vital capacity) (β = 9.51, SE = 2.95, *p* = 0.002). Additionally, the CC genotype of this polymorphism was associated with clinical manifestations of COVID-19 in COPD patients (χ^2^= 3,95, df = 2, *p* = 0.05). **Conclusions**: Our study identified a significant association between the *IREB2 rs2568494* polymorphism and an increased risk of severe COPD. The *IREB2 rs13180* polymorphism was linked to smoking behavior, as well as key lung function indicators, suggesting its potential role in disease progression and lung damage. Furthermore, the CC genotype of the *IREB2 rs13180* polymorphism was associated with clinical manifestations of COVID-19 in COPD patients, indicating a potential impact of this genetic variant on susceptibility to viral infections in this population.

## 1. Introduction

Chronic obstructive pulmonary disease (COPD) is a progressive respiratory condition characterized by persistent airway inflammation resulting from long-term exposure to hazardous gases and particles, such as cigarette smoke or air pollution. Recent studies suggest that the pathogenesis of COPD involves a complex interplay between genetic predisposition and environmental factors. Certain allelic combinations [1] define genetic factors that may enhance the negative impacts of environmental exposures, initiating and accelerating inflammatory and degenerative alterations in lung tissue.

Genome-wide association studies (GWASs) have demonstrated a strong association between COPD and polymorphisms in the *CHRNA3/5* (cholinergic nicotinic receptor alpha 3/5) and *IREB2* (iron-responsive element-binding protein 2) genes, both located within the chromosomal region 15q25 [2,3]. These genes encode proteins implicated in the regulation of inflammatory pathways and related to oxidative stress responses.

The 15q25 locus encompasses three genes encoding the α3, α5, and β4 subunits of the nicotinic acetylcholine receptor (nAChR), a key genomic region implicated in COPD susceptibility. Widely expressed in both neuronal and non-neuronal tissues, these receptors are essential for immune defense, mucociliary clearance, inflammation, and other processes [4]. nAChRs are also involved in the pathophysiology of several respiratory conditions, including asthma, COPD, and lung cancer [5]. Notably, the non-synonymous single-nucleotide polymorphism (SNP) *rs16969968* in *CHRNA5* has been strongly linked to smoking behavior and increased COPD risk [4]. Studies show that this variant increases susceptibility to oxidative stress, thereby contributing to lung tissue damage associated with COPD. *Rs16969968* is currently being investigated as a potential therapeutic target [6].

In addition to the cholinergic receptor genes, the *IREB2* gene within the same chromosomal region has also been linked to COPD pathogenesis [2]. Due to the high degree of linkage disequilibrium between *CHRNA5* and *IREB2*, distinguishing their individual effects remains challenging. *IREB2* encodes a protein involved in cellular iron metabolism by regulating iron-responsive elements (IREs) [7]. Disruption of iron homeostasis can lead to oxidative injury and localized pulmonary inflammation [8]. Previous studies, including gene expression analyses in lung tissues and meta-analyses, have reported associations between *IREB2* polymorphisms and increased COPD susceptibility [3].

Despite these findings, most studies exploring the genetic aspects of COPD have focused on European or East Asian populations [7,9,10,11,12]. Populations from Central Asia—including Kazakhstan—remain understudied, despite potentially distinct allele frequencies and environmental risk profiles. Populations from Central Asia, including Kazakhstan, remain poorly studied despite potentially different allele frequencies and environmental risk profiles. Therefore, this study aimed investigate whether specific variants in *CHRNA5* and *IREB2* are associated with COPD risk and phenotypic characteristics in a Kazakhstan population.

## 2. Materials and Methods

### 2.1. Subjects 

A total of 532 unrelated participants were enrolled in this study, including 265 patients diagnosed with COPD and 267 individuals in the control group, aged between 40 and 78 years. Participants were recruited between September 2022 and March 2024 at the Pulmonology Department and Respiratory Center of City Clinical Hospital No. 1, Almaty, Kazakhstan. Of the total cohort, 87.4% were of Kazakh origin, 7.3% were Russian, and 5.3% belonged to other ethnic groups, including Uighurs, Koreans, Tatars, and others. These efforts were made to compare case rates by age, sex, and ethnicity.

The inclusion and exclusion criteria were as follows: COPD diagnosis was established based on a comprehensive evaluation, including medical history, general and clinical assessments, special investigations (chest radiography, spirometry, computed tomography), physical examination, and laboratory tests according to the Global Initiative for Chronic Obstructive Lung Disease (GOLD, 2023) recommendations [13]. All COPD patients exhibited a postbronchodilator forced expiratory volume in 1 second (FEV_1_)/forced vital capacity (FVC) ratio of <70% and FEV_1_ < 80% of the predicted value to determine the disease stage. Exclusion criteria included a diagnosis of bronchial asthma, atopy, interstitial lung diseases, or lung cancer. Control subjects were included if they were aged 40 years or older, had normal pulmonary function (FEV_1_/FVC > 70%, FEV_1_ > 80%), and had no personal or family history of allergic or pulmonary diseases, including COPD. 

### 2.2. Genotyping

Genotyping was conducted at the Laboratory of Structural and Functional Genomics of the M. Aitkhozhin Institute of Molecular Biology and Biochemistry, Almaty, Kazakhstan. Blood samples (4 mL) were collected from COPD patients and controls. The ethnic origin (up to the third generation) of all participants was determined through direct interviews. Genomic DNA was isolated from blood via the DNeasy Blood & Tissue Kit (Qiagen, Germany) according to the manufacturer’s protocol. The quantity and quality of the isolated DNA were determined via a NanoVue Plus spectrophotometer (Richmond Scientific, UK).

Single-nucleotide polymorphisms (SNPs) were determined using the TaqMan™ SNP genotyping assay (ThermoFisher, Waltham, MA, USA, Catalog #4351379) at the following loci: *IREB2 rs13180* (assay ID C_8873396_1) and *IREB2 rs2568494* (assay ID C16043098_10), and *CHRNA5 rs16969968* (assay ID C26000428_20). PCR was performed in a 10 μl reaction volume under the conditions recommended by the manufacturer using TaqMan™ Genotyping Master Mix (ThermoFisher, Waltham, MA, USA, Catalog #4381656) on a StepOnePlus Real-Time PCR System (ThermoFisher, Waltham, MA, USA, Catalog #4376600). The results (quality control, determination of genotypes) were analyzed using the StepOnePlus v2.2.2 program (ThermoFisher, Waltham, MA, USA).

### 2.3. Statistical Analysis

Differences in baseline characteristics between study groups were evaluated via Student’s *t*-test. The χ^2^ test was used to evaluate deviations in genotype frequencies from the Hardy–Weinberg equilibrium in the control group. Allele and genotype frequencies were compared between cases and controls using odds ratios (ORs), 95% confidence intervals (CIs), and the Cochran–Armitage trend test. To assess associations between the SNPs and COPD, logistic regression analysis was performed with adjustments for age, sex, pack-years smoking, smoking status, and body mass index (BMI). Linear regression was used to evaluate the relationship between the SNPs and quantitative phenotypes, including smoking index and lung function parameters. Data analysis was carried out via SAS version 9 (SAS, Inc., Cary, NC, USA). Linkage disequilibrium (LD) structure in the *CHRNA3/5* and *IREB2* region and haplotypes were examined using Haploview 4.2 (Broad Institute of MIT and Harvard, Boston, MA, USA). Statistical significance was defined as *p* < 0.05.

## 3. Results

A total of 265 patients with severe chronic obstructive pulmonary disease (COPD) and 267 individuals in the control group were included in this study (Table 1). 

Both groups were predominantly male, and distributions of age, sex, and BMI were comparable between the groups. Patients with COPD exhibited a significantly higher mean smoking index (pack-years: 36.7 vs. 23.5, *p* < 0.005). Compared to COPD patients, control group participants demonstrated a significantly higher level of pulmonary function, as indicated by higher predicted FEV_1_ values (101.63% vs. 39.8%) and a higher FEV_1_/FVC ratio (0.82 vs. 0.59, respectively). Differences in smoking history and pulmonary function parameters were accounted for in both logistic and linear regression analyses.

We investigated the prevalence of polymorphic variants *rs13180*, *rs2568494*, and *rs16969968* in the *CHRNA3/5* and *IREB2* genes among COPD patients and controls (Table 2). 

No deviations from the Hardy–Weinberg equilibrium were observed for any of the genotyped SNPs in the control group. Based on the Cochran–Armitage trend test results (Table 2) and logistic regression analysis, no statistically significant differences in allele or genotype frequencies were identified between COPD patients and controls, either before or after adjustment for age, sex, BMI, smoking index, and current smoking status.

To investigate potential genetic determinants associated with more severe forms of COPD, patients with milder forms of the disease were excluded. The final study sample included patients with moderate, severe, and very severe COPD (corresponding to GOLD stages II–IV). 

Multivariate logistic regression analysis in this subset revealed a significant correlation (*p* = 0.03) between increased susceptibility to COPD and the AA genotype of the *IREB2 rs2568494* SNP (Table 3). 

This association remained statistically significant after adjustment for sex, age, BMI, pack-years, and smoking status. In contrast, neither the minor alleles of *CHRNA5 rs16969968* nor *IREB2 rs13180* showed statistically significant associations with COPD in this subset.

To assess potential gene–environment interactions, we conducted a stratified analysis by smoking status (never-smokers, former smokers, and current smokers) for evaluating the association between *IREB2* and *CHRNA5* gene polymorphisms and COPD. Multivariable logistic regression models accounted for age, sex, and BMI in the never-smoker group, and additionally for cumulative tobacco exposure (pack-years) in current and former smokers. After adjustment, none of the evaluated SNPs showed a statistically significant association with COPD in the never-smoker subgroup. However, the *IREB2 rs13180* variant was found to be significantly associated with COPD in both current and former smokers, suggesting a possible interaction between tobacco exposure and genetic susceptibility (Table 4).

The possible impact of the *IREB2* (*rs2568494* and *rs13180*) and *CHRNA5* (*rs16969968*) genotypes on pulmonary function and smoking-related behavior was evaluated using linear regression analysis. The smoking index, FEV_1_, and the FEV_1_/FVC ratio were dependent variables. In every model, age, sex, and BMI were included as covariates. The *IREB2 rs13180* variant was significantly associated with FEV_1_ (β = 7.79, SE = 2.98, *p* = 0.01) and FEV_1_/FVC (β = 9.51, SE = 2.95, *p* = 0.002). In particular, the T/T genotype was linked to a 9.51% increase in FEV_1_/FVC and a 7.79% increase in predicted FEV_1_, supporting the potential relevance of this polymorphism in lung function (Table 5).

Given that patient recruitment occurred during and shortly after the COVID-19 pandemic, COPD patients were classified into two groups: those with clinical manifestations and a confirmed diagnosis of COVID-19, and those with COPD who denied having contracted the virus. Preliminary data from this cohort have been previously reported by us [14]. Among patients with COVID-19 symptoms, the CC genotype of the *IREB2 rs13180* polymorphism was significantly more frequent (χ^2^ = 3.95, df = 2, *p* = 0.05) (Table 6).

No associations were found between the clinical manifestations of COVID-19 in COPD patients and the polymorphic variants *IREB2 rs2568494* and *CHRNA3/5 rs16969968* (χ^2^ = 1.72, df = 2, *p* = 0.19 and χ2 = 1.61, df = 2, *p* = 0.2, respectively). Despite their close physical proximity within the 15q25 region, the *IREB2* (*rs2568494* and *rs13180*) and *CHRNA3/5* (*rs16969968*) variants exhibited limited linkage disequilibrium (R^2^ = 0.21), suggesting that these SNPs may represent independent genetic signals. 

## 4. Discussion

Genetic predisposition plays a critical role in the development of COPD, with multiple genes contributing to disease susceptibility [1]. In this study, we investigated the associations of COPD with the *IREB2* (*rs2568494*, *rs13180*) and *CHRNA5* (*rs16969968*) genes in the Kazakhstan population.

The *IREB2* gene encodes the iron-binding protein 2 (IRP2), which is involved in maintaining cellular iron homeostasis [3]. IRP2 binds to iron-responsive elements (IREs) in mRNA, regulating the translation of genes critical for iron transport, storage, and utilization, such as TFRC (transferrin receptor) and FTH1 (ferritin) [15]. By modulating the balance between iron uptake and storage, IRP2 prevents toxic iron accumulation, which can lead to reactive oxygen species (ROS) production and subsequent cellular damage [16]. Iron is crucial for mitochondrial function and energy production [17], and *IREB2* indirectly influences these processes. Dysregulation of *IREB2* expression or function can result in imbalances in iron metabolism, contributing to oxidative stress and inflammation—key mechanisms in COPD pathogenesis and other diseases, such as neurodegenerative disorders and cancer [3,16,18].

Environmental factors such as smoking and cellular aging significantly affect iron accumulation in cells. Gio et al. demonstrated elevated iron levels in lung tissue among both healthy smokers and smokers with COPD than in nonsmokers [19]. This highlights tobacco smoke as a factor contributing to iron metabolism dysregulation. Studies have also shown that iron levels in lung tissue increase with age in humans and animal models (e.g., rats), which is potentially linked to the progression of inflammatory and degenerative changes in the lungs [20]. The interplay among age-related changes, smoking, and iron metabolism dysregulation underscores the complexity of genetic and environmental interactions in COPD pathogenesis.

The *CHRNA5* gene encodes the α5 subunit of the neuronal nicotinic acetylcholine receptor (nAChR), which can be activated by nicotine, the principal active compound among the 4000 chemicals in tobacco smoke, and plays a key role in the development of nicotine dependence [21]. The nAChR is a pentameric receptor that modulates the release of neurotransmitters such as dopamine, glutamate, and GABA. Studies have shown that genetic variants in the α-nAChR 3/5 subunit locus (CHRNA3/5) can influence nicotine dependence, smoking behaviors, and lung cancer risk [8,22,23].

The *rs16969968* polymorphism in *CHRNA5*, located at chromosome position 15q25.1, encodes the *CHRNA 5* subunit with a D398N modification (α5SNP) [24]. This variant is present in approximately 60% of the global population [2,25]. In our study, the minor allele A was observed at a frequency of 20.0% in COPD patients and 19.7% in controls. Previously, this SNP was independently associated with lung cancer [26], nicotine dependence [27], and COPD [25].

In our analysis, none of the selected SNPs showed significant associations with COPD, either before or after adjusting the models for age, sex, BMI, smoking history, and current smoking status. Given that prior studies identified associations predominantly in specific phenotypic subgroups, we conducted a stratified analysis by smoking status and excluded patients with mild disease (GOLD I). This approach revealed an association of *IREB2 rs2568494* with COPD stages GOLD II–IV. A significant association was identified for the *IREB2 rs13180* variant among both current and former smokers with COPD. Additionally, this genotype was associated with FEV_1_ and the FEV_1_/FVC ratio, as well as with clinical manifestations of COVID-19. In contrast, the *CHRNA5 rs16969968* polymorphism did not show a significant association with COPD or its phenotypic traits in our cohort.

These findings align with those reported in other studies. For instance, a study conducted in a Chinese Han population [10] reported no significant associations between SNPs in *IREB2* or *CHRNA3/5* and COPD via logistic regression. However, *IREB2 rs13180* was associated with FEV_1_% as predicted, and *rs16969968* in *CHRNA3/5* was linked to both FEV_1_% as predicted and FEV_1_/FVC in COPD patients. Similarly, an earlier study by Yi Guo et al. in the same population revealed no association between the *IREB2* variant *rs2568494* and COPD [28]. 

Despite these mixed results, numerous studies have demonstrated significant associations between polymorphic variants of the *IREB2* and *CHRNA5* genes and risk of COPD as well as impaired lung function across various populations.

In a study by Korytina et al. [12] involving patients with GOLD stages II–IV COPD, *rs13180* (*IREB2*) and *rs16969968* (*CHRNA5*) were significantly associated with COPD under an additive model. The CG haplotype of *rs13180* was identified as a protective factor for COPD in the Tatar population. Stratifying patients by smoking status confirmed associations with COPD risk for *rs16969968* (*CHRNA5*) in the additive model and *rs13180* (*IREB2*) in the recessive model. Additionally, *rs16969968* was linked to reduced FEV_1_ as a percentage of the predicted value. This study highlighted associations between *rs13180* and *rs16969968* and COPD and lung function in the Tatar population.

Megan Hardin et al. [9] investigated *rs13180* (*IREB2*) in a Polish population and found significant associations with severe COPD and related phenotypes, including lung function, smoking, and BMI. In contrast, a study by Ziółkowska-Suchanek et al. [29] examined associations of *IREB2* variants (*rs2568494* and *rs13180*) among 1141 participants (468 with lung cancer, 149 with COPD, and 524 smoking controls). While the minor *IREB2 rs2568494* AA genotype was more frequent in lung cancer patients than in controls, no significant associations of *rs2568494* or *rs13180* with COPD were observed. 

Chappell et al. [30] conducted a replication study in a cohort of white COPD patients (*n* = 1017) and healthy smoking controls (*n* = 912), confirming an association between *IREB2 rs2568494* and COPD. Similarly, a meta-analysis by Qiaoli Zeng et al. [8], including 4096 cases and 5870 controls, found that *rs2568494* was significantly associated with increased COPD risk under a dominant model.

Regarding *CHRNA5*, Hopkins RJ and colleagues [25] analyzed *CHRNA5 rs16969968*, concerning smoking exposure, lung function, and COPD status, in a large prospective cohort study including 9270 non-Hispanic white subjects. The high-risk AA genotype of *rs16969968* was associated with reduced lung function, increased smoking intensity, COPD presence, and lung cancer development. Furthermore, a comprehensive meta-analysis by Yang Lei et al. (2022) [31], involving 70,960 cases and 124,838 controls, revealed a significant association between the *rs16969968* polymorphism and COPD risk.

A study by Valencia-Pérez Rea D. et al. [32] explored the association between the *rs16969968* polymorphism (*CHRNA5*) and severe forms of COVID-19. The authors found that among COVID-19 patients who were smokers, those carrying the GA and AA genotypes exhibited elevated levels of erythrocyte sedimentation rate (ESR) and a positive correlation between fibrinogen and C-reactive protein, suggesting a potential role of this variant in modulating inflammatory responses. 

## 5. Conclusions

In conclusion, COPD arises from a complex interplay between genetic predisposition and environmental factors, whose lifelong interactions influence various biological mechanisms such as inflammation, apoptosis, and tissue repair, ultimately shaping the clinical manifestations of the disease. Genetic studies have highlighted the role of polymorphisms in the *IREB2* and *CHRNA5* genes in COPD susceptibility; however, their impact varies across populations, clinical phenotypes, and additional factors such as smoking.

Our findings are consistent with the existing literature, particularly regarding the significance of *rs2568494* in the *IREB2* gene among patients with GOLD II–IV COPD and emphasizing the absence of association for certain SNPs with COPD in the general cohort. The variations in the results between populations may be attributed to genetic diversity, phenotypic differences among patients, and variations in stratification methods.

Importantly, our analysis revealed a potential link between the *IREB2 rs13180* CC genotype and the presence of clinical manifestations of COVID-19 among COPD patients. This finding suggests that specific genetic variants may not only influence COPD susceptibility and severity, but also modulate the host response to respiratory viral infections such as SARS-CoV-2. These observations underscore the need for further research of gene–environment and gene–virus interactions in shaping disease outcomes in COPD, particularly in the context of emerging respiratory infections.

## Figures and Tables

**Table 1:** Characteristics of the main and control groups.

Variables	COPD (*n* = 265)	Controls (*n* = 267)	*p*
Male, *n* (%) Female, *n* (%)	182 (68.7%)	177 (66.3%)	
	83 (31.3%)	90 (33.7%)	
Age (mean ± SD) (year)	65.3 ± 9.9	64.3 ± 10.1	
BMI (mean ± SD) (kg/m^2^)	25.8 ± 6.3	27.8 ± 8.5	
Smoking status:			
Pack-years smoked (mean ± SD)	36.7 ± 17.1	23.5 ± 11.1	<0.05
Current and former smokers, *n* (%)	176 (66.4%)	148 (55.4%)	<0.001
Non-smokers, *n* (%)	89 (33.6%)	119 (44.3%)	<0.001
Post-FEV1% (mean ± SD)	39.8 ± 16.7	101.63 ± 14.8	<0.0001
Post-FEV1/FVC ratio (mean ± SD)	0.59 ± 0.27	0.82 ± 0.45	<0.0001
GOLD stage (%):			
I, *n* (%) II, *n* (%) III, *n* (%) IV, *n* (%)	37 (13.9%)		
	58 (21.9%)	-	
	106 (40.0%)	-	
	64 (24.2%)	-	

BMI, body mass index; SD, standard deviation; FEV_1_, forced expiratory volume in 1 second; FVC, forced vital capacity; GOLD, Global Initiative for Obstructive Lung Disease.

**Table 2:** Characteristics of SNPs, allele frequencies, and genotype distributions of *CHRNA5* and *IREB2* polymorphisms in COPD patients and control individuals.

SNP	Chromosome Position (NCBI)	Category	Minor Allele	Alleles/Genotypes	HWE *p* Value in Controls	COPD Patients (%) *n* = 265	Controls (%) *n* = 267	OR (95% CI)	*p*
*IREB2: rs2568494*	76528019	Intron	A	G	0.51	368 (69.4)	384 (71.9)	0.89 (0.68–1.16)	0.38
				A		162 (30.6)	150 (28.1)	1.13 (0.87–1.47)	
				G/G		130 (49.1)	135 (50.6)	0.94 (0.67–1.32)	0.37
				G/A		108 (40.8)	114 (42.7)	0.92 (0.65–1.30)	
				A/A		27 (10.2)	18 (6.7)	0.57 (0.84–2.92)	
*IREB2: rs13180*	76576543	Exon	C	T	0.46	269 (50.8)	278 (52.1)	0.96 (0.75–1.21)	0.67
				C		261 (49.2)	257 (47.9)	1.05 (0.83–1.34)	
				T/T		74 (27.9)	68 (25.5)	1.13 (0.77–1.67)	0.67
				T/C		121 (45.7)	142 (53.2)	0.74 (0.53–1.04)	
				C/C		70 (26.4)	57 (21.3)	1.32 (0.89–1.97)	
*CHRNA5: rs16969968*	76669980	Exon	A	G	0.45	424 (80.0)	429 (80.3)	0.98 (0.72–1.32)	0.89
				A		106 (20.0)	105 (19.7)	1.02 (0.76–1.38)	
				G/G		168 (63.4)	175 (65.5)	0.91 (0.64–1.30)	0.89
				G/A		88 (33.2)	79 (29.6)	1.18 (0.82–1.71)	
				A/A		9 (3.4)	13 (4.9)	0.69 (0.29–1.64)	

SNP, single-nucleotide polymorphism; NCBI, National Center for Biotechnology Information; HWE, Hardy–Weinberg equilibrium; OR, odds ratio; CI, confidence interval.

**Table 3:** Genotype frequencies of *CHRNA5* and *IREB2* polymorphisms in patients with COPD (stages II–IV) and control individuals.

SNP	Genotypes	COPD Patients (%) N = 228	Controls (%) N = 267	ORadj	95% CIadj	*p* Value	*p* Value adj
*IREB2: rs2568494*	G/G	97 (42.5)	135 (50.6)	1.00		0.08	0.03 *
	G/A	105 (46.1)	114 (42.7)	0.27	0.08–0.94		
	A/A	26 (11.4)	18 (6.7)	0.69	0.23–2.10		
*IREB2: rs13180*	T/T	57 (25.0)	68 (25.5)	1.00		0.3680	0.3773
	T/C	113 (54.8)	142 (53.2)	0.58	0.26–1.25		
	C/C	58 (20.2)	57 (21.3)	0.72	0.37–1.39		
*CHRNA3/5: rs16969968*	G/G	136 (59.6)	175 (65.5)	1.00		0.6017	0.327
	G/A	86 (37.7)	79 (29.6)	2.75	0.69–10.92		
	A/A	6 (2.6)	13 (4.9)	2.81	0.72–10.95		

SNP, single-nucleotide polymorphism; OR, odds ratio; CI, confidence interval; * *p* ≤ 0.05.

**Table 4:** Association analysis of IREB2 and CHRNA5 SNPs with COPD in smoking-status stratified groups.

Gene/SNP	Nonsmokers (*n* = 89)	Former Smokers (*n* = 112)	Current Smokers (*n* = 64)
*IREB2 rs2568494*	0.60	0.63	0.73
*IREB2 rs13180*	0.52	0.02 *	0.05 *
*CHRNA 3/5 rs16969968*	0.98	0.65	0.72

SNP, single-nucleotide polymorphism; * *p* ≤ 0.05.

**Table 5:** Evaluation of the influence of the *IREB2* (*rs2568494* and *rs13180*) and *CHRNA5* (*rs16969968*) genes on the smoking index, FEV_1_, and FEV_1_/FVC.

	*IREB2 rs2568494*			*IREB2 rs13180*			*CHRNA5 rs16969968*		
	Standard Error	*t* Value	*p* Value	Standard Error	*t* Value	*p* Value	Standard Error	*t* Value	*p* Value
Smoking index (Pack-Years)	28.09	−0.21	0.84	11.59	−0.91	0.36	26.67	0.86	0.39
FEV_1_	7.22	−0.04	0.97	2.98	2.61	0.01 *	6.86	−1.72	0.09
FEV_1_/FVC	7.15	0.79	0.43	2.95	3.22	0.002 *	6.79	−1.42	0.16

* *p* ≤ 0.05.

**Table 6:** Genotype frequencies of *CHRNA5* and *IREB2* polymorphisms in COPD patients with and without clinical manifestations of COVID-19 during the pandemic.

SNP	Genotypes	COVID-19 (+) *n* = 87, (%)	COVID-19 (-) *n* = 178, (%)	OR	95% CI	*p* Value
*IREB2: rs2568494*	G/G	54 (62.1)	86 (48.3)	1.75	1.04–2.95	
	G/A	25 (28.7)	81 (45.5)	0.48	0.28–0.84	
	A/A	8 (9.2)	11 (6.2)	1.54	0.60–3.97	0.19
*IREB2: rs13180*	T/T	30 (34.5)	48 (27.0)	1.43	0.82–2.48	
	T/C	22 (25.3)	105 (59.0)	0.24	0.13–0.42	
	C/C	35 (40.2)	25 (14.0)	4.12	2.26–7.52	0.05 *
*CHRNA3/5: rs16969968*	G/G	62 (71.3)	110 (61.8)	1.53	0.88–2.67	
	G/A	19 (21.8)	54 (30.3)	0.64	0.35–1.17	
	A/A	6 (6.9)	14 (7.9)	2.81	0.32–2.34	0.2

SNP, single-nucleotide polymorphism; OR, odds ratio; CI, confidence interval; COVID-19 (+) and COVID-19 (−)—COPD patients with and without COVID-19 symptoms; * *p* ≤ 0.05.

## Data Availability

The data presented in this study are available on request from the corresponding author. The data are not publicly available due to privacy and ethical issues.
